# Supplementary material for: Evidence of Extensive Circulation of Yersinia enterocolitica in Rodents and Shrews in Natural Habitats from Retrospective and Perspective Studies in South Caucasus
Source: Pathogens. 2021 Jul 26;10(8):939. doi: 10.3390/pathogens10080939 (PMC8400892; doi:10.3390/pathogens10080939)
Supplement: Supplementary file 1 [file pathogens-10-00939-s001.zip › pathogens-1297493-supplementary.pdf]

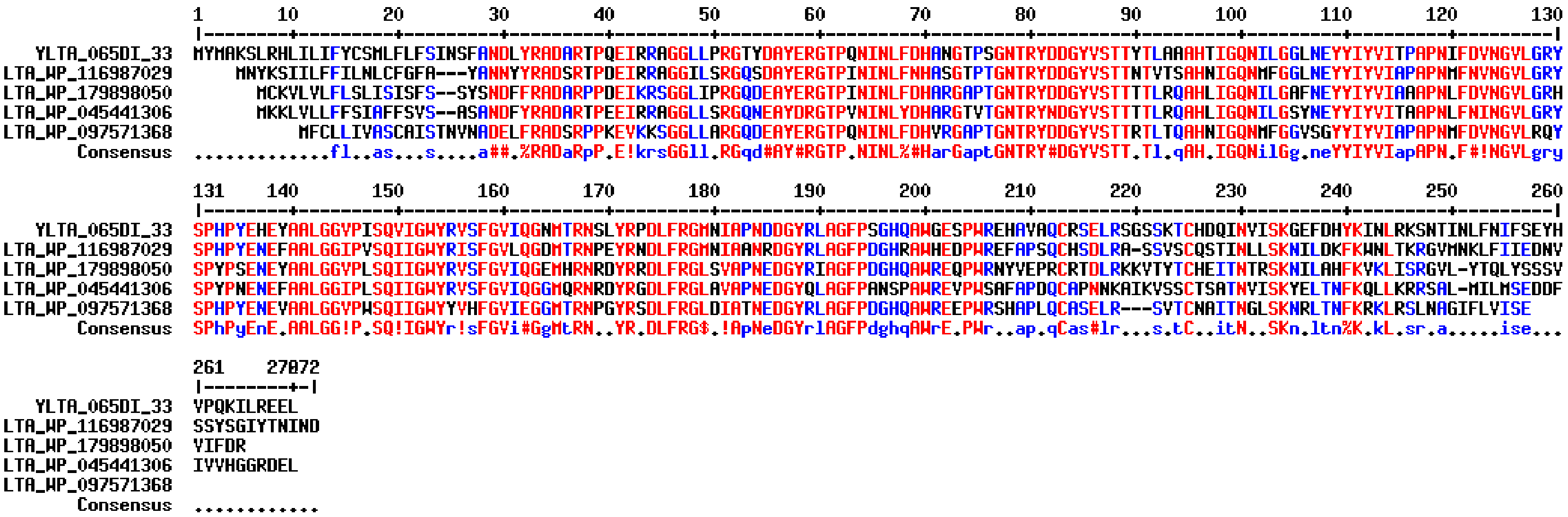

Figure S1. Alignment of heat-labile enterotoxin, A chain (YLTA) of *Y. enterocolitica* 18E17 065 D I -33 (YLTA_065DI_33) with LTA homologs from other microorganisms deposited in GenBank, such as *E. coli* (WP_116987029, 66/80%), *Providencia alcalifaciens* (WP_179898050, 68/80%), *Cronobacter* (WP_097571368, 62/75%), and *Citrobacter sp.* S-77 (WP_045441306, 60/76%). GenBank accession numbers for these LTAs, as well as percent of their identity/similarity with YLTA are shown in parentheses. The aligment was visualized in MultAlin (http://multalin.toulouse.inra.fr/multalin/).

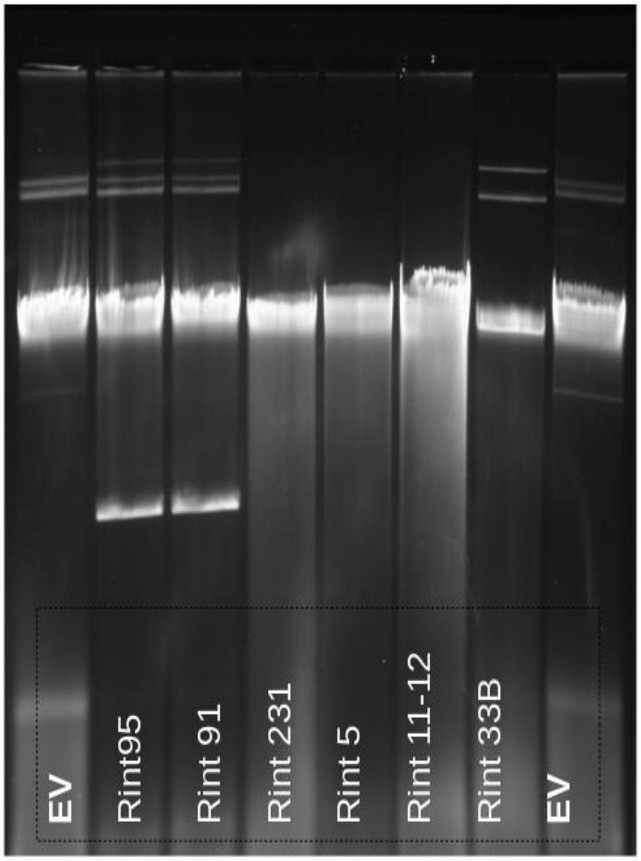

Figure S2. Plasmid profiles of the strains obtained by Kado & Liu method. *Yersinia pestis* EV76 (EV) vaccine strain containing reference plasmids with molecular weight 101, 70.5, and 9.5 kb. *Y. enterocolitica* strains 18E17 065 D I Rint-95 (Rint95), 18E17 065 D I Rint-91 (Rint91), B18 Rint -231 (Rint231), LcENCDC Rint 5 (Rint5), 18ENCDC AsAtc Rint - 11-12 (Rint11-12), and 18E17 065 D I -33 (Rint33B).

```
                   1       10        20        30        40        50        60        70        80        90        100       110       120       130
                   |--------+---------+---------+---------+---------+---------+---------+---------+---------+---------+---------+---------+---------|
      ORF1_Rint95  MAEGNVNTGFFVKFNNTIMDAINKMGDTYQAEYATGIMSLMVGSITLYILYVGYSTLAGKTTTPVPDLVWNLARFAIITAFISNAGGYLTSLSEGVQGLKDGFSGGTSVWATLDTLWAGTQDLATDVFQR
VirB6_WP_011191407        MAGFFEKFNTTIMDKLTTMGDVYQAQYATGIMSLMVGAVTLYILYVGYSTLGGKLQTPVPDLVWNLARFAMLITFITNAGGYLTALTEGLNGLKAGFTGDTSVWAALDTLWDSTQKLADKIYDK
virB6_WP_050292434        MAGFFEKFNTTVMDKLTTMGDVYQAQYATGIMSLMVGAVTLYILYSGYSTLAGKQQTPVPDLVWNLARFAMLITFITNAGGYLSALTDGLNGLKTGFTGDTSVWATLDTLWASTQELADKIYDK
virB6_WP_172986724        MAGFFEKFNTTVMDKLTTMGDVYQAQYATGIMSLMLGAVTLYILWVGYSSLAGKIQTPVPDLVWNLARFAMLITFITNAGGYLTSLTEGLNGLKAGFTGNTSVWATLDTLWASTQELADKIYDK
   virB6_STT63835      MAGQGFFVKFNNTIMDSVQKISDSYQSQFASDIMTLAAASVTLYVLWKGYQTLAGKTQTPLPDLAWDLTKFAIIITFITNAGGYLTSATTAMEGIKDGLSGGVSVWQTLDNLWASTQNLAEKIYQK
  TrbL_WP_001473361         MSFFVKYNTTVMEAIDKVGSTYQSQFTQDIMSLVTASVTLYVLWKGYQILASKTQTPMQDLVWDLSKFAIIIMFITNADGYLTAATNALQGMKDGFSGGVSVWQTLDNLWEKTQNLAKKVYQK
   TriE_EBJ6215970     MAG--FFVKFNNELMTKLEQIGQTYQSQFASDIMTLATASVTLYVLWKGYQTLAGKTQTPLPDLAWDLAKFAIIITFITNAGGYLTSATEALKGVKDGLSGGVSVWQTLDNLWASTQAISEKLYQI
        Consensus  ........gFFvK%Nnt.Mdkl..mgdtYQa#%atgIMsLm.gs!TLY!Lw.GYstLagKtqTPvpDLvW#LarFAiiitFItNAgGYLtsltegl.GlKdGfsGgtSVWatLDtLWasTQ.la.k.%#k

                   131     140       150       160       170       180       190       200       210       220       230       240       250       260
                   |--------+---------+---------+---------+---------+---------+---------+---------+---------+---------+---------+---------+---------|
      ORF1_Rint95  DDS-WVPVLGWLGMLCIWGGSIVLMITSAVVFLTADLTMKFMLITAPIFLFCLMFGFLRPMFNNWLQLIFSSILTVLFASMIINLAVDFQIDIADQLRAGVDDGNLMTMGAMGGVAGLLAAVLVTLAAGF
VirB6_WP_011191407 DKSTYVAVSGGLGAALVWGGAIFLMFTSAVVFLTADITMLFMLMTAPIFLFCLMFGFLRPMFNSWLQLIFSSILTVLFASIVIRLAIDFQVEIAGQVATQADDSNLMTMGAMGAVAGLLSGVLVTLAAGF
virB6_WP_050292434 DESTYVAVSGGIGSALVWAGAIFLMFTSAVVFLTADMTMLFMLMTAPIFLFCLMFGFLRPMFNNWLQLIFSSILTVLFASIVIRLAIDFQVEIAGQVAAQADDKNLMTMGAIGAVSGALSGVLVTLAAGF
virB6_WP_172986724 DESTYVAVSGGFGAALVWAGAIFLMFTSAVVFLTADMTMLFMLMTAPIFLFCLMFGFLRPMFNNWLQLIFSSILTVLFASIIIRLAIDFQVDIAGQVATQANDKNLMTMGAMGAVSGLLSGVLVTLAAGF
   virB6_STT63835  DTD-YIPLAGAFGMALVWAGSMILMAISTVVYLAADVTMKLLIITAPIFIFCLMFGFLRQMFNNWLQLNFSSILTVLFASLVIRIGMDYQGEILQQVSNAAPNSNVVTAGAMGFMAGLLSALLVMLARMF
  TrbL_WP_001473361  DTD-FIPLAGALGMTLVWAGSLILMTISAVVFLTADITMKFLIITAPIFIFCLMFGFIRVMFNNWLQSLFSSILTVLFASLVIRIAMDFQGDILQQVSNATPNSNVMTVSAMGFLAGLLGALLVLIAKSF
   TriE_EBJ6215970  DAD-MVPLAGFFGSACVWAGSIILMAVSSVVFISADVVMTLMIITAPIFLFCLMFGFLRQMFNNWLQTVFSSILTVLFASLVIRFCMDFQGDMLTQVLKGADRPNLFTAGAMGLVAGILSSILVVVSSKI
        Consensus  D.s..!pv.G..G.al!WaGsi.LM.tSaVV%ltAD.tM.f$liTAPIFlFCLMFGFlRpMFNnWLQliFSSILTVLFAS.!Irla.D%Q.#ia.Qv...add.NlmTmgAmG.vaGlLs.vLVtlaagf

                   261     270       280       290       300       310       320       330       340       350       360       370  375
                   |--------+---------+---------+---------+---------+---------+---------+---------+---------+---------+---------+----|
      ORF1_Rint95  ASKLAGAGAEGAIQGMAMAGIMKAAKPIAKAAAKGAGKAAGAAGKALGSIGDKAASNLQGAAPVNPAAARAKAAVESMKAYTASQ------SRK
VirB6_WP_011191407 ASKLAGAGAEGAMQGLAMAGAMKAAKPVGKAAAKGAGKVAGGIGNGLGGIGNKAASNLNSAPAVNPTAARAKAAVESMKAFSASQ------SSKIVSK
virB6_WP_050292434 ASKLAGAGAEGAMQGLAMAGAIKVAKPAGKSAAKGTGKAAGAAAGAAGNIATKAGDAAYSAMN-SPAKVRAKAAVEAMKTYTSSQ------S-KIVSK
virB6_WP_172986724 AQRLAGAGAEGAVQGMANAGIISGGKLAGRSAKPIAEGVKGA-----NSLAEKTGEAAYSAYN-SPAKVRAKAAVEAMKTYTASQ------S-KIVSK
   virB6_STT63835  ATQLAGAGVDGAVQGMAMMGLGAAGVGASRLGMSGGKAGFGLMKGLTGKDVGNTASNSSRLGNFLGRGTRSASEWAGQKMQGADAWAGQKIQSGINSMADRRRASLAKKRSEAA
  TrbL_WP_001473361  AVQLAGAGVEGAVQGAAMMGLGAAGMATGKSLMLGGRAGLGFGLGMAGRTGMN--SLSGKAGNLMGRGARTAAEWAGEKGYQALA------PTGALGMKARRLASLEKARARNAA
   TriE_EBJ6215970  AMSLAGAGVDGAVQGMAVLGVGASGKAAASLGSVGLRSGVGLGMGLAGKKGMQ--SFAGKAGNAVGRGTHSAAEWMGDRAYQKMA------STGVVGAKARRLASLEKARARNAA
        Consensus  A..LAGAGa#GAvQGmAmaG...agk.a.k.a..g.....G...g..g..g.k..s....a.n..p...rakaave.mkay.asq......s.k.v..................
```

Figure S3. Alignment of ORF1 of small plasmid (Contig_23, 4,543 bp) of *Y. enterocolitica* 18E17 065 D I Rint-95 (ORF1_Rint95) with the type 4 secretion system VirB6 conjugal transfer protein from the microorganisms deposited in GenBank, such as *Yersinia pseudotuberculosis* (WP_011191407, 74/83%), *Yersinia kristensenii* (WP_050292434, 70/82%), *Yersinia massiliensis* (WP_172986724, 66/78%), *Klebsiella pneumoniae* NCTC9632 (STT63835, 57/75%), *E.coli* (WP_001473361, 54/70%), and *Salmonella enterica* PNUSAS047895 (EBJ6215970, 51/67%). GenBank accession numbers for these proteins, as well as percent of their identity/simililarity with the ORF1_Rint95 are shown in parentheses. The aligment was visualized in MultAlin (http://multalin.toulouse.inra.fr/multalin/).

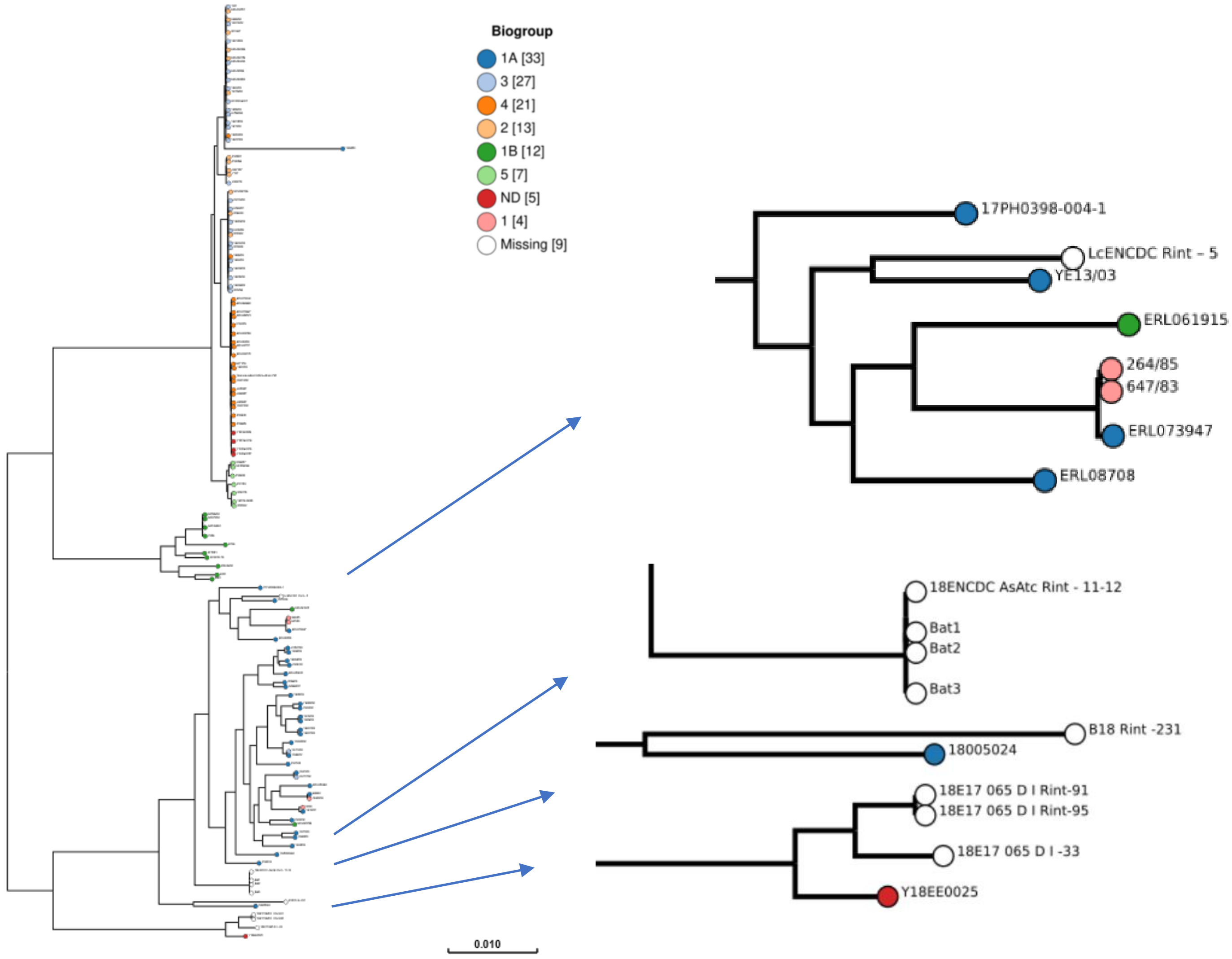

Figure S4.

Maximum likelihood- phylogenetic SNP-based tree constructed in Enterobase showing the position of sequenced *Y. enterocolitica* isolates from Georgia in the context of worldwide *Y. enterocolitica* biogroup distribution. These Georgian *Y. enterocolitica* isolates were 18E17 065 D I Rint-95, 18E17 065 D I Rint-91, B18 Rint -231, LcENCDC Rint 5, 18ENCDC AsAtc Rint - 11-12, and 18E17 065 D I -33. Previously described *Y. enterocolitica* isolates from dead bats (Imnadze et.al., 2020) designated as Bat1, Bat2 and Bat3. The tips are colored according to the biogroups of the isolates.

Table S1. *Yersinia enterocolitica* strains phenotypic characterization by API20E

No	Strain ID	ONPG Beta-Galactosidase	ADH Arginine DiHydrolase	LDC Lysine DeCarboxylase	ODC Onithin DeCarboxylase	CIT Citrate Simmons	H2S Production	URE Urease	TDA Tryptophan DesAminase	IND Indole	VP Voges Proskauer	GEL Gelatine Hydrolyse	GLU Glucose	MAN Manitol	INO Inositol	SOR Sorbitol	RHA Rhamnose	SAC Sucrose	MEL Meliboise	AMY Amygladin	ARA Arabinose
1	18ENCDC AsAtc Rint 11-12	neg	neg	neg	pos	neg	neg	pos	neg	pos	pos	neg	pos	pos	pos	pos	neg	pos	neg	pos	pos
2	LcENCDC Rint - 5	neg	neg	neg	neg	neg	neg	pos	neg	pos	pos	neg	pos	pos	pos	pos	neg	pos	neg	neg	neg
3	18E17 065 D I -33	neg	neg	neg	pos	neg	neg	pos	neg	pos	pos	neg	pos	pos	pos	pos	neg	pos	neg	pos	pos
4	18E17 065 D I Rint-95	neg	neg	neg	pos	neg	neg	pos	neg	pos	pos	neg	pos	pos	pos	pos	neg	pos	neg	pos	pos
5	18E17 065 D I Rint-91	neg	neg	neg	pos	neg	neg	pos	neg	pos	pos	neg	pos	pos	pos	pos	neg	pos	neg	pos	pos
6	B18 Rint -231	pos	neg	neg	pos	neg	neg	pos	neg	pos	pos	neg	pos	pos	pos	pos	neg	pos	neg	pos	pos
7	Lc18Encdc Rint -2	pos	neg	neg	neg	neg	neg	pos	neg	pos	pos	neg	pos	pos	pos	pos	neg	pos	neg	pos	pos
8	LcEncdc Rint - 4	neg	neg	neg	neg	neg	neg	pos	neg	pos	pos	neg	pos	pos	pos	pos	neg	pos	neg	neg	neg
9	18E17 065 D I -36	neg	neg	neg	pos	neg	neg	pos	neg	pos	pos	neg	pos	pos	pos	pos	neg	pos	neg	pos	pos
10	18E17 065 D I -86	pos	neg	neg	pos	neg	neg	pos	neg	pos	pos	neg	pos	pos	pos	pos	neg	pos	neg	pos	pos
11	18E17 065 D I -29	pos	neg	neg	pos	neg	neg	pos	neg	pos	pos	neg	pos	pos	pos	pos	neg	pos	neg	pos	pos

12	18E17 065 D I -108	neg	neg	neg	pos	neg	neg	pos	neg	pos	pos	neg	pos	pos	pos	pos	neg	pos	neg	pos	pos
13	18E17 065 D I Rint-63	pos	neg	neg	pos	neg	neg	pos	neg	pos	pos	neg	pos	pos	pos	pos	neg	pos	neg	pos	pos
14	18E17 065 D I Rint-88	neg	neg	neg	pos	neg	neg	pos	neg	pos	pos	neg	pos	pos	pos	pos	neg	pos	neg	pos	pos
15	18E17 065 D I Rint-107	pos	neg	neg	neg	neg	neg	pos	neg	pos	pos	neg	pos	pos	pos	pos	neg	pos	neg	pos	pos
16	18EAs P0094 I Rint - 8	pos	neg	neg	pos	neg	neg	pos	neg	pos	pos	neg	pos	pos	pos	pos	neg	pos	neg	pos	pos
17	18EAs P0094 I Rint - 4	pos	neg	neg	pos	neg	neg	pos	neg	pos	neg	neg	pos	pos	pos	pos	neg	pos	neg	pos	pos
18	18EAs P0094 I Rint - 19	pos	neg	neg	pos	neg	neg	pos	neg	pos	neg	neg	pos	pos	pos	pos	neg	pos	neg	pos	pos
19	18EAs P0094 I Rynt - 9	pos	neg	neg	pos	neg	neg	pos	neg	pos	pos	neg	pos	pos	pos	pos	neg	pos	neg	pos	pos
20	18ESachxP0094 I Rint - 8	pos	neg	neg	pos	neg	neg	pos	neg	pos	pos	neg	pos	pos	pos	pos	neg	pos	neg	pos	pos
21	18ESachxP0094 I Rint - 14	pos	neg	neg	pos	neg	neg	pos	neg	pos	neg	neg	pos	pos	pos	pos	neg	pos	neg	pos	pos
22	18ESachxP0094 I Rint - 7	pos	neg	neg	neg	neg	neg	pos	neg	pos	pos	neg	pos	pos	pos	pos	neg	pos	neg	pos	pos
23	18ESachxP0094 I Rint - 22	neg	neg	neg	pos	neg	neg	pos	neg	pos	pos	neg	pos	pos	pos	pos	neg	pos	neg	pos	pos
24	18ESachxP0094 I Rint - 25	neg	neg	neg	pos	neg	neg	pos	neg	pos	pos	neg	pos	pos	pos	pos	neg	pos	neg	pos	pos
25	18ESachxP0094 I Rint - 30	neg	neg	neg	neg	neg	neg	pos	neg	pos	pos	neg	pos	pos	pos	pos	neg	pos	neg	neg	neg
26	18E17 065 D II Rint -33	neg	neg	neg	neg	neg	neg	pos	neg	pos	pos	neg	pos	pos	pos	pos	neg	pos	neg	neg	neg
27	18E17 065 D II Rint-36	neg	neg	neg	neg	neg	neg	pos	neg	pos	pos	neg	pos	pos	pos	pos	neg	pos	neg	neg	neg

28	18E17 065 D II Rint-106	neg	neg	neg	pos	neg	neg	pos	neg	pos	pos	neg	pos	pos	pos	pos	neg	pos	neg	pos	pos
29	18E17 065 D II Rint -14	pos	neg	neg	pos	neg	neg	pos	neg	pos	pos	neg	pos	pos	pos	pos	neg	pos	neg	pos	pos
30	18E17 065 D II Rint-104	pos	neg	neg	pos	neg	neg	pos	neg	pos	pos	neg	pos	pos	pos	pos	neg	pos	neg	pos	pos
31	18Encdc Poti Rint-3	neg	neg	neg	pos	neg	neg	pos	neg	pos	pos	neg	pos	pos	pos	pos	neg	pos	neg	pos	pos
32	18E Tsk P0094 Rint - 5	neg	neg	neg	neg	neg	neg	pos	neg	pos	pos	neg	pos	pos	pos	pos	neg	pos	neg	neg	neg
33	18E17 065 D III Rint -198	neg	neg	neg	pos	neg	neg	pos	neg	pos	pos	neg	pos	pos	pos	pos	neg	pos	neg	pos	pos
34	B18 Rint -246	pos	neg	neg	pos	neg	neg	pos	neg	pos	pos	neg	pos	pos	pos	pos	neg	pos	neg	pos	pos
35	B18 Rint -263	neg	neg	neg	pos	neg	neg	pos	neg	pos	pos	neg	pos	pos	pos	pos	neg	pos	neg	pos	pos
36	B18 Rint -247	pos	neg	neg	pos	neg	neg	pos	neg	pos	neg	neg	pos	pos	pos	pos	neg	pos	neg	pos	pos
37	18E17 065 D III Rint -327	neg	neg	neg	neg	neg	neg	pos	neg	pos	pos	neg	pos	pos	pos	pos	neg	pos	neg	neg	neg
38	18Encdc Lag Rint - 4	neg	neg	neg	neg	neg	neg	pos	neg	pos	pos	neg	pos	pos	pos	pos	neg	pos	neg	neg	neg
39	18Encdc Lag Rint - 18	neg	neg	neg	pos	neg	neg	pos	neg	pos	pos	neg	pos	pos	pos	pos	neg	pos	neg	pos	pos
40	19E Rxv BAA Axm Rint - 58	pos	neg	neg	pos	neg	neg	pos	neg	pos	pos	neg	pos	pos	pos	pos	neg	pos	neg	pos	pos
41	19E Rxv BAA Axm Rint - 65	pos	neg	neg	pos	neg	neg	pos	neg	pos	pos	neg	pos	pos	pos	pos	neg	pos	neg	pos	pos
42	19E Rxv BAA Axm Rint - 71	neg	neg	neg	neg	neg	neg	pos	neg	pos	pos	neg	pos	pos	pos	pos	neg	pos	neg	neg	neg
43	19E17 065 D IV Rint -24	neg	neg	neg	pos	neg	neg	pos	neg	pos	pos	neg	pos	pos	pos	pos	neg	pos	neg	pos	pos

44	19E17 065 D IV Rint -28	neg	neg	neg	neg	neg	neg	pos	neg	pos	pos	neg	pos	pos	pos	pos	neg	pos	neg	neg	neg
45	19E Kh P0094 Rint - 26	neg	neg	neg	neg	neg	neg	pos	neg	pos	pos	neg	pos	pos	pos	pos	neg	pos	neg	neg	neg
46	19E Kh P0094 Rint - 6	pos	neg	neg	pos	neg	neg	pos	neg	pos	pos	neg	pos	pos	pos	pos	neg	pos	neg	pos	pos
47	19E Ambr P0094 II Rint - 28	pos	neg	neg	pos	neg	neg	pos	neg	pos	pos	neg	pos	pos	pos	pos	neg	pos	neg	pos	pos
48	19E Ambr P0094 II Rint - 27	neg	neg	neg	neg	neg	neg	pos	neg	pos	pos	neg	pos	pos	pos	pos	neg	pos	neg	neg	neg
49	19E Ambr P0094 II Rint - 37	neg	neg	neg	pos	neg	neg	pos	neg	pos	pos	neg	pos	pos	pos	pos	neg	pos	neg	pos	pos
50	19E Ambr P0094 II Rint - 35	neg	neg	neg	pos	neg	neg	pos	neg	pos	pos	neg	pos	pos	pos	pos	neg	pos	neg	pos	pos
51	19E Ambr P0094 II Rint - 34	neg	neg	neg	neg	neg	neg	pos	neg	pos	pos	neg	pos	pos	pos	pos	neg	pos	neg	neg	neg
52	19E Ambr P0094 II Rint - 46	neg	neg	neg	neg	neg	neg	pos	neg	pos	pos	neg	pos	pos	pos	pos	neg	pos	neg	neg	neg
53	19E Ambr P0094 II Rint - 52	pos	neg	neg	pos	neg	neg	pos	neg	pos	neg	neg	pos	pos	pos	pos	neg	pos	neg	pos	pos

Table S2. Presence of the classical virulence-associated genes of *Yersinia enterocolitica* in the strains from Georgia sequenced in this study.

Gene/ Function	18E17 065 D I Rint-91 & 18E17 065 D I Rint-95	B18 Rint - 231	18E17 065 D I -33	LcENCDC Rint 5	18ENCDC AsAtc Rint - 11-12
ystB/ Heat-stable enterotoxin type B	+	+	+	+	+
inv/ Invasin	+	+	+	–	–
myfA/ Fimbrial protein MyfA	–	+	–	+	+
ymoA/ Modulator of expression for virulence functions	+	+	+	+	+
ytxA/ ADP-ribosyltransferase Pertussis-like toxin	+	+	–	+	+
hreP (*prcA*)/ In vivo-expressed subtilisin/kexin-like protease	–	+	–	+	+
fepBDGC/ Ferric enterobactin transport system	+	+	+	+	+
rtxA/ RtxA-like putative leukotoxin	–	+	–	+	+
cdtB/ Cytolethal distending toxin subunit B-like protein	–	+	–	+	+
hemPR-hmuVSTU/ Direct heme uptake system	+	+	+	+	+
yax/ pore-forming cytotoxin Cytotoxin YaxAB	+	+	+	+	+
phlA (*yplA*)/ Phospholipase A homolog	+	+	+	+	+
pldA/ Phospholipase A1	+	+	+	+	+
flg/ Flagella cluster I	+	+	+	+	+
arsCBR/ Arsenic cluster	+	+	+	+	+
yts1/ Type 2 secretion	+	+	+	+	+
spiA, ssaV-ssaN/ Chromosomal Type 3 secretion system	+	–	+	+	+
virF/ Virulence regulon	–	–	–	–	–

transcriptional activator					
pYV plasmid/ Virulence plasmid encoding Type 3 secretion	–	–	–	–	–
yltA/ heat-labile enterotoxin, A chain	–	–	+	–	–
ystA/ Heat-stable Enterotoxin type A	–	–	–	–	–
ail/ Attachment invasion locus protein (adhesin)	–	–	–	–	–
vapBC/ type II toxin–antitoxin systems	+	+	+	+	–
vapC/ tRNA(fMet)-specific endonuclease VapC	+	–	+	–	–
tccC/ Insecticidal toxin	–	–	–	–	–
plasmids	4	0	2	0	0

The plus and minus signs represent presence or absence of the genes.
